# Complete genome sequence of Baby Chick Ranikhet Disease Vaccine (BCRDV) strain in Bangladesh

**DOI:** 10.1128/mra.00681-25

**Published:** 2025-09-17

**Authors:** Nasreen Parveen, Md. Rezaul Karim, Shushoma Swaraj Happy, Tahmina Begum, Anowar Hossen, ASM Ashab Uddin, Tania Ahmed, Sultana Jahan, Md. Mostofa Kamal, Mohammed A. Samad

**Affiliations:** 1 Department of Livestock Services, Livestock Research Institute (LRI) 560480, Dhaka, Bangladesh; 2 Genome Sequence Laboratory, Transboundary Animal Disease Research Center, Bangladesh Livestock Research Institute (BLRI) 560480, Savar, Dhaka, Bangladesh; DOE Joint Genome Institute, Berkeley, California, USA

**Keywords:** complete genome sequence, Newcastle disease virus, BCRDV vaccine

## Abstract

Newcastle disease (ND), a highly contagious viral disease of poultry, is commonly controlled using live-attenuated vaccines. The BCRDV vaccine in Bangladesh was developed from the lentogenic strain of ND virus through serial passaging in embryonated chicken eggs. Here, we present the complete genome sequence of the BCRDV vaccine strain used in Bangladesh.

Newcastle disease (ND) is an acute and highly contagious viral disease of poultry caused by Newcastle disease virus (NDV) or avian paramyxovirus type 1 (APMV-1), a negative-sense, single-stranded RNA virus of the genus *Orthoavulavirus* of the family *Paramyxoviridae* (1). NDV exhibits neurological, respiratory, gastrointestinal, and reproductive signs, especially in unvaccinated birds (2). The genome encodes six structural proteins, with the fusion (F) protein, which determines virulency (3). NDV strains are categorized into lentogenic or mild, mesogenic or moderate, and velogenic or very virulent pathotypes. Biosecurity and vaccination, especially using live and inactivated or genotype-matched vaccines, are essential for prevention and control of ND (4).

Here, we report the complete genome sequence of the BCRDV vaccine strain (GenBank accession no. PV809488). The complete genome sequence was determined using next-generation sequencing (NGS) on the Illumina platform, by aligning it with the complete genome sequence of NDV reference sequence (NC_075404.1). Total RNA was extracted from the culture supernatant of the master seed of BCRDV vaccine strain in 9–10-day-old embryonated chicken eggs using the Quick-RNA Viral Kit (Cat. No. R1035, Zymo Research), following the manufacturer’s protocol. A sequencing library was prepared using the Illumina Standard Total RNA Prep, Ligation with Ribo-Zero Plus kit, resulting in an average fragment size of 400 bp. Sequencing was performed on the Illumina NextSeq 2000 platform with 2 × 150 bp paired-end chemistry, yielding approximately 1.62 million read pairs. Raw read quality was assessed using FastQC v0.11.3 (5). Adapter trimming and quality filtering were conducted with Trimmomatic v0.39.2 (6), applying the following parameters: minimum quality score >30, minimum read length >35 bp, and 5′ clip of 15 bases for both forward (R1) and reverse (R2) reads. Reads specific to BCRDV were identified using Kraken2 v2.1.3 (7) in conjunction with the viral reference database (version: k2_viral_20210517). The extracted BCRDV reads were assembled *de novo* using SPAdes v3.14.0 with k-mer sizes of 77 and 99 (8). The resulting assembly was manually curated to confirm the final viral genome sequence with median depth coverage, 1,971×, which was determined to be 15,186 bp in length, containing six open reading frames. The presence of six open reading frames was identified using Prokka annotation. Completeness of the genome was confirmed by alignment with the NDV reference sequence (NC_075404.1) in GenBank, which showed full coverage of all coding sequences (CDS) as well as the non-coding 5′ and 3′ UTRs, indicating that sequencing extended to both genome ends. The average GC content of the genome was 46%. Genome annotation was initially performed using Prokka v1.14.6 (9), and annotations were manually refined before submission to NCBI (10).

Sequence analysis revealed that the complete genome of the vaccine strain, designated BCRDV, is 15,186 nucleotides (nt) in length, comprising six transcriptional units in the order 3′-NP-P-M-F-HN-L-5′, with respective gene lengths of 1,470 nt (NP), 1,188 nt (*P*), 1,095 nt (M), 1,662 nt (F), 1,719 nt (HN), and 6,615 nt (L). Multiple sequence alignment of the deduced amino acid sequences was conducted to assess genetic and antigenic features of our study sequence in comparison with reference NDV strains retrieved from GenBank, including LaSota (JF950510), Hitchner-B1 (AF309418), Komarov (KT445901), and Herts/33 (AY741404). Analysis of the fusion (F) protein cleavage site demonstrated the presence of the motif ^112^G-R-Q-G-R↓L^117^, which is characteristic of lentogenic NDV strains, confirming the low-virulence nature of the BLRI-LRI-BCRDV vaccine strain. WebLogo analysis of the deduced amino acid sequences further supported the multiple sequence alignment findings. The monobasic amino acid at the C terminus of the cleavage site and leucine residue at 117 suggest low pathogenicity. In BLASTn analysis, the nucleotide of BLRI-LRI-BCRDV sequence shows 99.69% identity with the NDV strain from Croatia (KJ670427.1), 99.66% identity with the lentogenic NDV strain F from Bangladesh (ON713865.1), and 99.62% identity with the ndv59/F strain from India (KM056355.1) in the NCBI Core nucleotide database (core_nt). A phylogenetic tree was constructed using the maximum likelihood method coupled with the Kimura two-parameter model with bootstrap analysis of 100 replicates in MEGA, version X (11). Phylogenetic analysis showed that the BCRDV vaccine strain clusters with other representative lentogenic pathotypes (Fig. 1). The VaxiJen server (12) also indicated that the BCRDV strain possesses higher antigenic potential compared to its corresponding standard pathotype strains, with an overall protective antigen prediction score of 0.5620.

**Fig 1:**
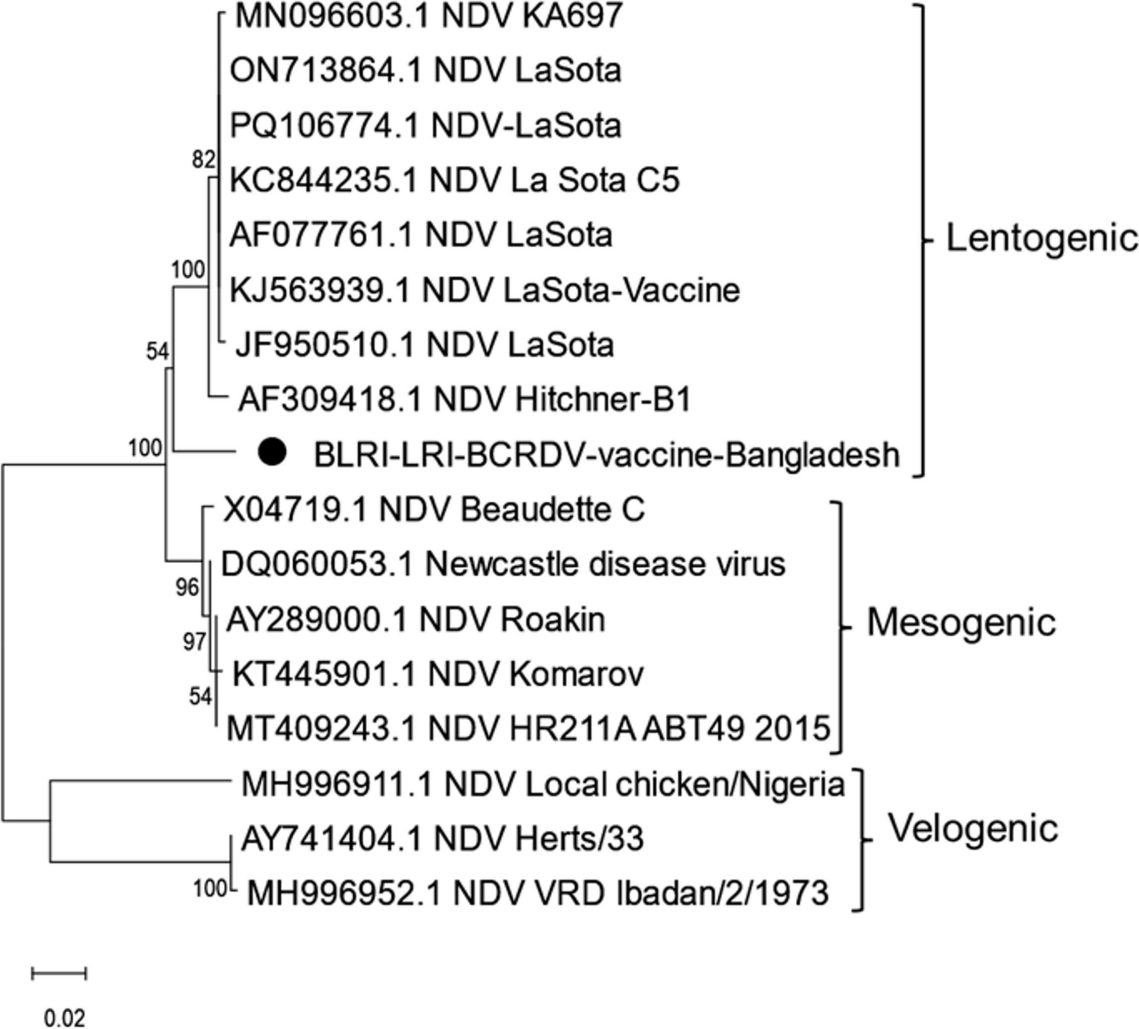
Phylogenetic analysis of BLRI-LRI-BCRDV vaccine complete nucleotide sequences. The tree was analyzed by maximum likelihood method with bootstrapping (100). Bar, 0.02 nucleotide substitutions per site. Sequences from this study are depicted with black circles.

## Data Availability

The complete genome sequence of the BCRDV vaccine strain in Bangladesh has been submitted to GenBank under accession number PV809488. The raw sequence reads have been submitted to the SRA database under accession number SRR34089964.
